# Investigations of Procalcitonin, Interleukin-8 and Defensin-β in Dogs with Superficial and Deep Pyoderma

**DOI:** 10.3390/vetsci13020183

**Published:** 2026-02-12

**Authors:** Stephan Neumann, Maren Dölle

**Affiliations:** 1 Institute of Veterinary Medicine, Georg-August University of Goettingen, Burckhardtweg 2, D-37077 Goettingen, Germany; 2 AniCura Tierärztliche Spezialisten Hamburg, Rodigallee 85, D-22043 Hamburg, Germany

**Keywords:** pyoderma, dog, PCT, IL-8, Defb2

## Abstract

**Background:** Canine pyoderma is a common bacterial skin disease that can be classified as superficial or deep and is associated with inflammatory processes. Systemic inflammatory biomarkers such as procalcitonin (PCT), interleukin-8 (IL-8), and beta-defensin-2 (Defb2) may reflect immune activation; however, their diagnostic and clinical relevance in canine pyoderma remains unclear. **Materials and Methods:** Serum concentrations of PCT, IL-8, and Defb2 were measured using enzyme-linked immunosorbent assays (ELISA) in healthy control dogs (group 1, *n* = 40), dogs with superficial pyoderma (group 2a, *n* = 16), and dogs with deep pyoderma (group 3a, *n* = 7). A subset of dogs with superficial pyoderma (group 2b, *n* = 12) was re-evaluated after clinical remission. Biomarker concentrations were statistically compared between groups and over time. **Results:** Dogs with superficial and deep pyoderma exhibited significantly higher serum concentrations of PCT and IL-8 compared to healthy controls, whereas Defb2 concentrations were significantly reduced in both disease groups. No statistically significant differences were detected between superficial and deep pyoderma for any of the biomarkers, although IL-8 showed a trend toward higher concentrations in dogs with deep pyoderma (*p* = 0.07). Follow-up examinations after clinical improvement revealed no significant changes in biomarker concentrations. **Conclusions:** Canine pyoderma is associated with measurable systemic inflammatory alterations, characterized by increased serum concentrations of PCT and IL-8 and decreased Defb2 levels, irrespective of disease depth. The lack of biomarker normalization following clinical remission suggests that systemic inflammatory responses may persist beyond visible clinical healing. While these biomarkers may provide complementary information on inflammatory activity, their utility for monitoring treatment response appears limited.

## Simple Summary

This study investigated serum concentrations of three inflammatory biomarkers—procalcitonin (PCT), interleukin-8 (IL-8), and beta-defensin-2 (Defb2)—in dogs with superficial and deep pyoderma and compared them to healthy control dogs. Dogs affected by pyoderma showed significantly higher serum concentrations of PCT and IL-8, while Defb2 concentrations were significantly lower than in healthy controls, indicating a systemic inflammatory response associated with bacterial skin infection. No significant differences in biomarker concentrations were detected between superficial and deep pyoderma. In dogs re-examined after clinical improvement, biomarker levels remained largely unchanged. These findings suggest that clinical resolution of pyoderma does not necessarily coincide with normalization of systemic inflammatory markers.

## 1. Introduction

Inflammation is an essential reaction of the organism to various triggers with the aim of restoring tissue integrity. The inflammatory process is well orchestrated over various stages under the influence of different cells and mediators. In addition to the classic inflammatory parameters of leukocyte count and differentiation, other parameters such as acute phase proteins have become established [1]. Furthermore, newer parameters may generate additional information, which is why some of these parameters were investigated in this study. The selection took into account the course of a general inflammatory reaction and generated markers from the different phases of inflammation.

PCT is a key marker of the systemic inflammatory response and is increasingly released, particularly in the early phase of a bacterial infection, to activate immune cells and control the inflammatory response [2,3]. IL-8 plays a central role in the middle inflammatory phase by recruiting neutrophil granulocytes as a strong chemokine, while Defb2 contributes to the elimination of pathogens and the maintenance of the epithelial barrier in the late phase through its antimicrobial effect.

In detail: PCT is a 116-amino acid polypeptide and the prohormone of calcitonin, which under physiological conditions is predominantly synthesized in the C-cells of the thyroid gland [4]. While under physiological circumstances this process leads to rapid conversion into active calcitonin, systemic bacterial infections result in extrathyroidal expression of PCT, including in the liver, lungs and kidneys [5]. In these cases, the prohormone is not further processed, which leads to increased PCT serum concentrations. Although the exact molecular mechanisms have not yet been fully elucidated, proinflammatory cytokines such as interleukin-6 (IL-6), interleukin-1 (IL-1) and tumor necrosis factor-α (TNF-α) are considered to be central mediators of this induction [4].

IL-8 is a pro-inflammatory chemokine that plays a key role in controlling leukocyte migration. It binds to the receptors CXCR1 and CXCR2 on neutrophils and other immune cells and thus enables their directed migration into inflamed tissue [5,6,7]. In addition to its role as a chemoattractant, IL-8 also activates neutrophils, which leads to the release of antimicrobial enzymes and thus contributes to an effective defense against infection [8].

In addition, IL-8 influences angiogenesis and tissue regeneration by stimulating the growth of new blood vessels [9]. Due to these versatile functions, IL-8 is not only considered a marker for inflammatory processes, but also an active regulator within complex immunological reactions [10].

Defensins are small cationic peptides that are produced by granulocytes, epithelial cells and other immune cells and stored in intracellular granules. After activation, they are released and develop their antimicrobial effect as part of the innate immune response [11,12]. A distinction is mainly made between α- and β-defensins, which differ structurally and with regard to their mechanism of action [13].

The antimicrobial activity is primarily based on the interaction with negatively charged membrane lipids of microbial cells. Defensins bind to these structures, destabilize the cell membrane by forming pores and ultimately lead to the cell death of the pathogens [14].

In addition to their effect against bacteria and fungi, defensins also exhibit antiviral properties by neutralizing viral particles or preventing their entry into host cells [15].

Pyoderma in dogs is a bacterial skin infection predominantly caused by *Staphylococcus pseudintermedius*, with pathogenesis rooted in complex cellular mechanisms involving bacterial virulence factors and host immune evasion. Predisposing factors include allergic dermatitis, endocrinopathies, and ectoparasitic infestations, which compromise epidermal integrity and immune surveillance [16]. Clinical manifestations range from superficial pustules and epidermal collarettes to deep nodules, ulceration, and furunculosis [17]. 

Cellular pathogenesis initiates with bacterial adherence mediated by cell wall-associated surface proteins (SpsD, SpsL), which bind fibrinogen, fibronectin, and cytokeratin 10, facilitating colonization of mammalian skin [18]. Biofilm formation constitutes a critical virulence mechanism, with over 90% of clinical isolates producing robust polysaccharide biofilms encoded by the *ica* operon (icaADBC) [19]. Biofilms confer antimicrobial resistance, impede immune cell penetration, and facilitate chronic infection by protecting bacteria within an extracellular polymeric matrix [19]. 

Dogs with pyoderma show elevated serum antistaphylococcal IgG levels compared to healthy dogs [20]. C-reactive protein (CRP) is significantly increased in dogs with pemphigus foliaceus, exceeding levels found in superficial pyoderma [21,22]. Deep pyoderma is associated with marked immunosuppression, characterized by decreased T lymphocyte and phagocyte activity [23,24]. 

The aim of the present studies was to investigate the influence of superficial and deep pyoderma on systemic inflammatory processes, measured by the above-mentioned markers PCT, IL-8 and as a representative for the defensins Defb2. Furthermore, it should be found out whether the markers can be measurable parameters for the severity of the skin inflammation and can therefore be used as progression parameters.

## 2. Materials and Methods

### 2.1. Animals

A total of 63 dogs, patients of the Institute of Veterinary Medicine, University of Goettingen and AniCura Tierärztliche Spezialisten Hamburg between 1 September 2020 and 1 June 2024, were examined in the present study and divided into three different groups. Group 1 were clinically and dermatologically healthy dogs (*n* = 40). Group 2a/b were dogs with superficial pyoderma (*n* = 28; 2a = 16; 2b = 12) and group 3 patients with deep pyoderma (*n* = 7). The dogs were of different breeds, with mixed breeds being the most common. The age of the dogs was between one and 16 years. The overall gender distribution was nearly balanced (32 male; 31 female).

Dogs in the healthy control group 1 showed no evidence of any disease on clinical, dermatological and laboratory examination (hematology and clinical biochemistry). Patients who had been treated in any way in the eight weeks prior to the study, e.g., with antibiotics, NSAIDs or corticoids, were not included in this group. Group 2a was defined by the presence of one or more of the following clinical signs: papules, pustules, epidermal collarettes or crusts. At least one neutrophil containing intracytoplasmic bacteria was detected on cytological examination, and the microbiology result was positive. For microbiology Bacterial isolates were cultured on standard bacteriological media, and species identification was performed using matrix-assisted laser desorption/ionization time-of-flight mass spectrometry (MALDI-TOF MS) (Bruker Optik GmbH, Leipzig, Germany). Group 3 consisted of patients with at least one additional sign, such as dermal nodules, skin fistulas or cellulitis, plus the detection of neutrophils with intracytoplasmic bacteria on cytology and a positive microbiology result.

The severity of pyoderma in both groups was assessed according to the literature [25]. The severity of each sign (papules, pustules, epidermal collarettes, crusts, dermal nodules, skin fistulas or cellulitis) was rated from 0 to 4. Dogs with a total score of less than 10 were assessed as having no to mild symptoms, those with a score of 10–20 were assessed as having moderate symptoms, and those with a score greater than 20 were assessed as having severe symptoms.

Dogs in groups 2 and 3 were monitored according to the severity index, with the biomarkers being re-examined after a decrease in severity in group 2 (group 2b). Group 3 was too small for re-examination.

All investigations were officially approved by the state authority for animal welfare (Laves, Postfach 3949, 26029 Oldenburg) under Az33.9-42502-05-16A089.

### 2.2. Sample Collection and Preparation

The EDTA and serum blood samples were taken, before any other procedure, from the cephalic veins with a 20-gauge needle (Sarstedt AG, Nümbrecht, Germany). Laboratory tests of hematology and serum biochemistry were performed using a ProCyte Dx Haematology Analyser (IDEXX GmbH, Kornwestheim, Germany) and a Konelab 20i analyzer (Thermo Fisher Scientific, Frankfurt/Dreieich, Germany). On hematology leucocytes, erythrocytes were counted, the packed cell volumen was maesured and the leucocytes were differentiated. On clinical biochemistry, urea, creatinine, phosphorus, glucose, albumin, total protein, cholesterol, Alanin transaminase, Asparta transaminase, Creatinin kinase, sodium, potassium and calcium were measured.

The blood samples for the analysis of PCT, IL-8 and Defb2 were allowed to clot for two hours at room temperature, then centrifuged at 1000× *g* for 20 min and the serum was collected. All serum samples for the analysis of PCT, IL-8 and Defb2 were stored at −80 °C until further analysis.

### 2.3. Quantitative Enzyme-Linked Immunosorbent Assay (ELISA)

The concentrations of PCT, IL-8 and Defb2 were quantified with commercially available canine-specific quantitative sandwich ELISA kits. The PCT was determined with the Canine Procalcitonin ELISA-Kit, ECP0215 (ABclonal, Woburn, MA, USA), IL-8 was measured with Canine Interleukin 8 ELISA Kit, MBS045317 (MyBioSource, San Diego, CA, USA) and Defb2 was measured using the Canine Defensin Beta 2 ELISA Kit, MBS028858 (MyBioSource, San Diego, CA, USA). Briefly, assay reagents were at room temperature before analysis. The procedure started with the addition of 100 μL sample or standard to the reaction wells and then 100 μL PBS buffer and 50 μL enzyme solution were added and incubated at room temperature. After washing the reaction wells, 50 μL substrate was added and after a second incubation period of 10 to 15 min 50 μL stop solution was added. The absorbance was measured with a TECAN multi-detection microplate reader (TECAN Austria GmBH, Grödig, Austria) at a wavelength of 450 nm. The ELISA kit’s inter-assay (%) coefficients of variation (CV) were less than 15% for PCT, IL-8 and Defb2.

### 2.4. Statistical Analysis

Statistical analyses were performed using commercially available software (Prism 10, GraphPad Software Inc., San Diego, CA, USA). Data distribution was assessed using the Shapiro–Wilk test. As most variables were not normally distributed, data are presented as median (minimum–maximum). Group differences between healthy dogs, dogs with superficial pyoderma, and dogs with deep pyoderma were analyzed using the Mann–Whitney test, followed by post hoc pairwise comparisons where appropriate. For graphical visualization, biomarker concentrations were displayed on a logarithmic scale. For longitudinal analyses, follow-up measurements within the same individuals were compared using the Wilcoxon signed-rank test. Changes in biomarker concentrations over time were additionally evaluated in relation to changes in clinical severity and cytological scores. Associations between serum biomarker concentrations and clinical severity index or cytological score were assessed using Spearman's rank correlation. In addition to absolute biomarker concentrations, ratios between biomarkers (e.g., IL-8/procalcitonin) were calculated and analyzed to explore potential improvements in diagnostic or inflammatory discrimination. Receiver operating characteristic (ROC) curve analysis was performed to evaluate the diagnostic performance of selected biomarkers and biomarker ratios for differentiation between superficial and deep pyoderma, as well as for the identification of high cytological scores. A *p*-value ≤ 0.05 was considered statistically significant.

## 3. Results

### 3.1. Superficial Pyoderma

All 16 dogs included in Group 2a presented with clinical signs consistent with superficial pyoderma. The initial severity scores ranged from 7 to 20, with a mean value of 14, indicating a moderate to severe clinical presentation at baseline. Treatment was individualized according to the assessed severity score and consisted of standard therapeutic measures for superficial bacterial skin infections.

Follow-up sampling of serum biomarkers was scheduled to monitor the inflammatory response under treatment. Follow-up examinations were performed after a mean treatment duration of 14 days. At re-evaluation (Group 2b), the severity index ranged from 0 to 5, with a mean value of 2, reflecting a marked clinical improvement in the majority of dogs.

Microbiological examination revealed *Staphylococcus pseudintermedius* as the causative pathogen in all dogs with superficial pyoderma. In one dog, a secondary bacterial species (*Escherichia coli*) was additionally identified.

### 3.2. Deep Pyoderma

All seven dogs included in Group 3 presented with clinical signs consistent with deep pyoderma. The initial severity scores ranged from 11 to 23, with a mean value of 17, indicating a generally more severe clinical presentation compared to dogs with superficial pyoderma. Therapeutic management and clinical monitoring were guided by the individually assessed severity scores. Microbiological analysis identified Staphylococcus pseudintermedius in all dogs with deep pyoderma. In addition, each dog was affected by at least one further bacterial species. Identified co-pathogens included *Streptococcus canis*, *Pasteurella multocida*, and *Escherichia coli*, indicating a polymicrobial infection pattern characteristic of deep pyoderma.

### 3.3. Serum Biomarker

All serum biomarker concentrations were assessed for normal distribution using the Shapiro–Wilk test. None of the three biomarkers showed a normal distribution in any of the study groups (Shapiro–Wilk, *p* < 0.05). Therefore, biomarker concentrations are presented as median (med), minimum (min), and maximum (max).

### 3.4. PCT Serum Concentrations

**Figure 1:**
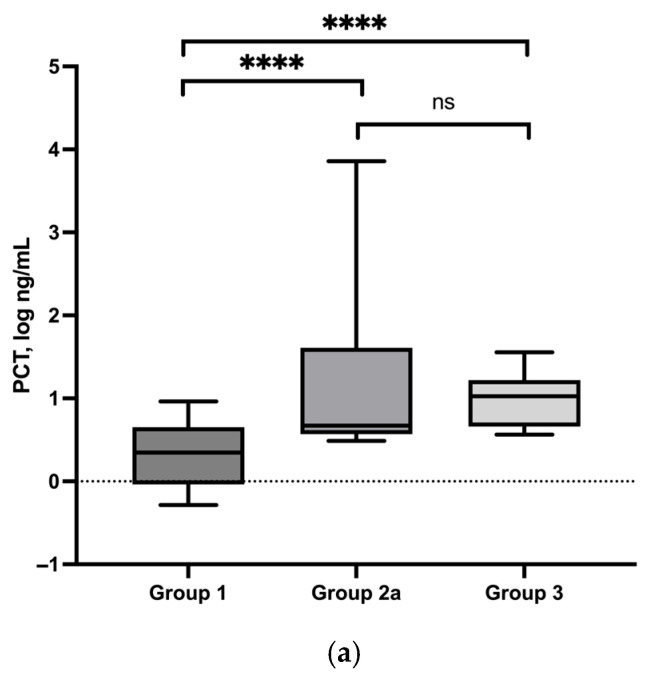

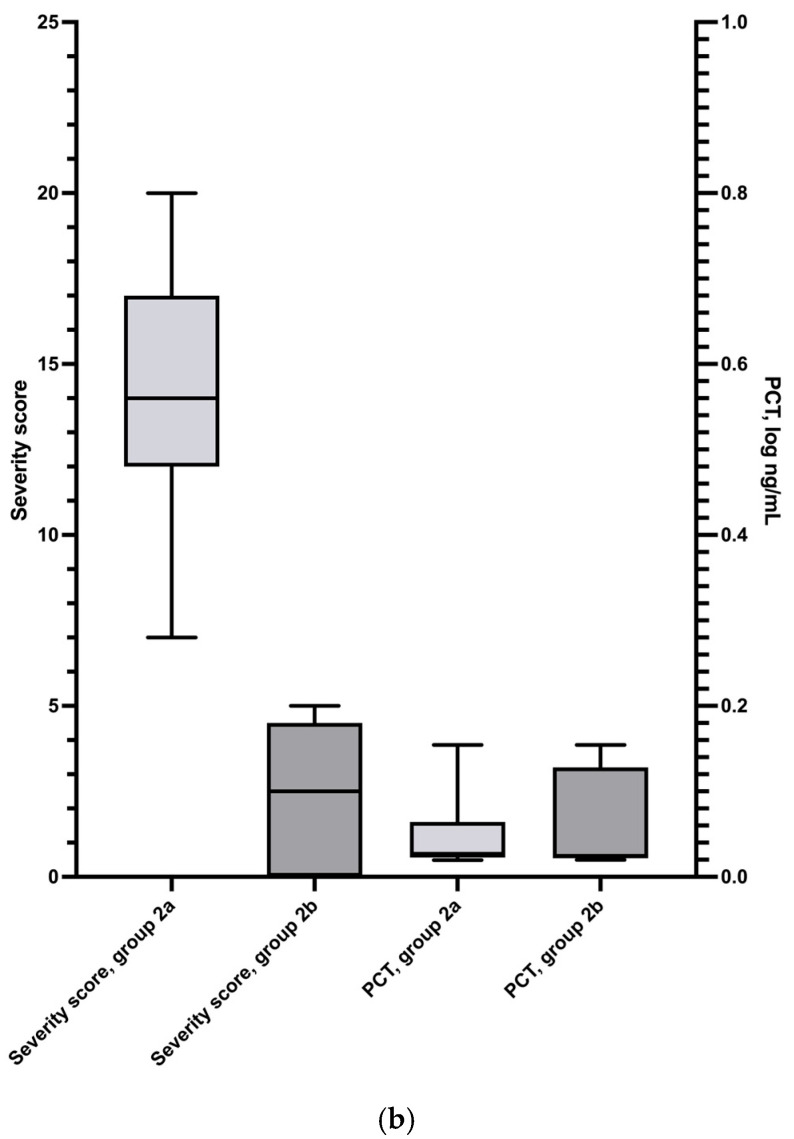
(**a**) PCT serum concentrations (log) of control group (group 1), dogs with superficial pyoderma (group 2a) and deep pyoderma (group 3) and their statistical comparison. ns = not significant, **** < 0.0001. (**b**) Comparison between changes in severity and serum PCT concentrations (log) between groups 2a and 2b. While severity shows a marked improvement at follow-up, serum PCT concentrations remain unchanged.

In the control group (Group 1, *n* = 40), PCT serum concentrations were determined in 39 dogs, with a median concentration of 2.2 ng/mL (min/max: 0.5–9.1 ng/mL) (Table 1). Dogs with superficial pyoderma (Group 2a, *n* = 16) showed higher PCT serum concentrations, with a median value of 4.7 ng/mL (min/max: 3.0–7228.0 ng/mL). Comparison between all study groups revealed a significant increase in PCT concentrations in dogs with superficial pyoderma compared to healthy controls (*p* < 0.0001). In dogs with deep pyoderma (Group 3, *n* = 7), PCT serum concentrations were also significantly higher than in healthy controls, with a median concentration of 10.6 ng/mL (min/max: 3.6–35.9 ng/mL; *p* < 0.0001) (Figure 1a). In contrast, PCT serum concentrations did not differ significantly between dogs with superficial pyoderma (Group 2a) and dogs with deep pyoderma (Group 3) (*p* > 0.4). In a subset of dogs with superficial pyoderma (Group 2b, *n* = 12), follow-up measurements were performed after clinical remission (median severity index: 14 → 2). At follow-up, median PCT serum concentrations were 4.1 ng/mL (min/max: 3.1–7244.0 ng/mL) and did not differ significantly from baseline values (*p* = 0.8) (Figure 1b).

### 3.5. IL-8 Serum Concentrations

**Figure 2:**
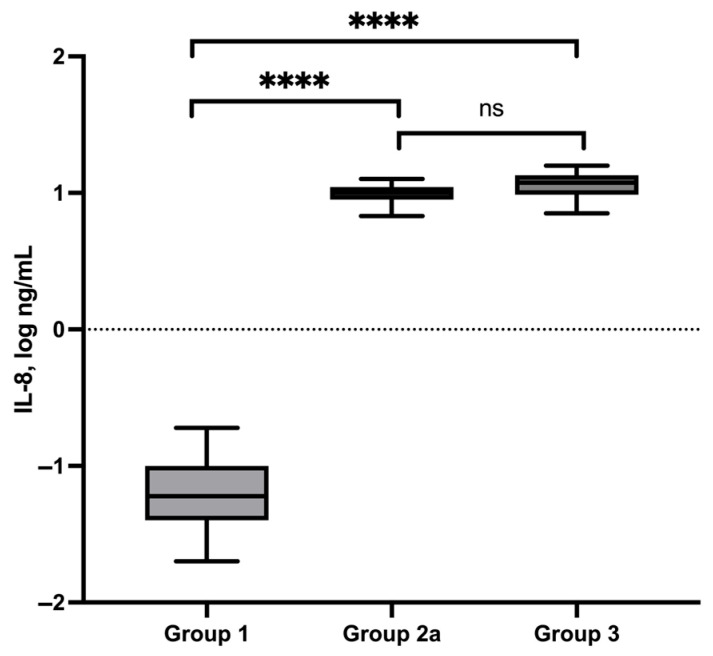
IL-8 serum concentrations (log) of control group (group 1), dogs with superficial pyoderma (group 2a) and deep pyoderma (group 3) and their statistical comparison. ns = not significant, **** <0.0001.

IL-8 serum concentrations were measured in 33 dogs of the control group (Group 1). The median concentration was 0.06 ng/mL (min/max: 0.02–0.19 ng/mL) (Table 1). Dogs with superficial pyoderma (Group 2a) exhibited markedly increased IL-8 serum concentrations, with a median value of 10.0 ng/mL (min/max: 6.8–12.6 ng/mL). In dogs with deep pyoderma (Group 3), the median IL-8 serum concentration was 11.9 ng/mL (min/max: 7.1–15.9 ng/mL). IL-8 concentrations in both pyoderma groups were significantly higher compared to healthy controls (*p* < 0.0001). No statistically significant difference was detected between dogs with superficial and deep pyoderma, although a trend toward higher concentrations in dogs with deep pyoderma was observed (*p* = 0.07) (Figure 2). In follow-up examinations after clinical remission (Group 2b, *n* = 12; severity index: 14 → 2), IL-8 serum concentrations remained largely unchanged compared to baseline values (median: 10.3 ng/mL; min/max: 7.9–16.2 ng/mL). No statistically significant difference between baseline and follow-up measurements was observed (*p* = 0.48).

**Figure 3:**
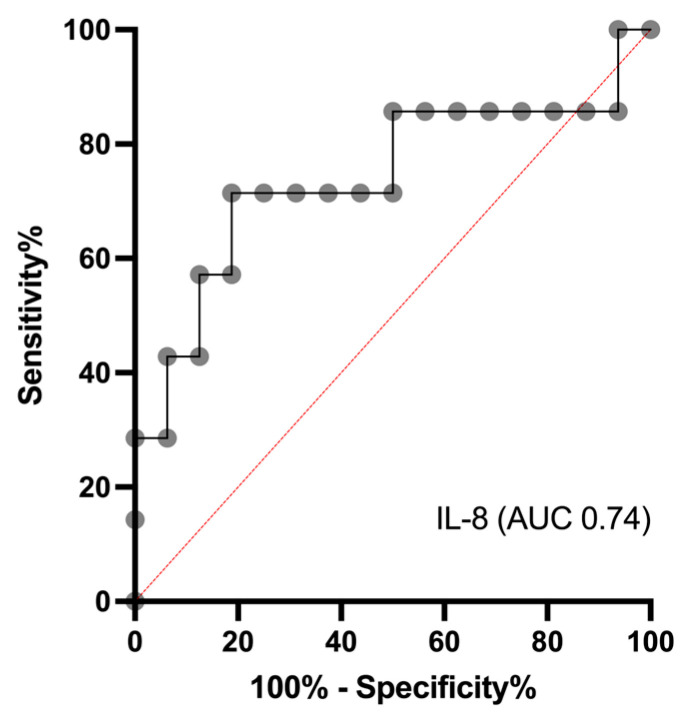
Receiver operating characteristic (ROC) analysis of interleukin-8 (IL-8). An IL-8 serum concentration of 11.0 ng/mL represented the optimal cut-off value for differentiating superficial from deep pyoderma, yielding a sensitivity of 0.71 and a specificity of 0.79. The area under the curve (AUC) was 0.74.

Beyond evaluating differences in serum concentrations between diagnostic groups, the clinical applicability of inflammatory biomarkers depends on their ability to distinguish between disease entities at the individual patient level. Receiver operating characteristic (ROC) analysis is a widely used method for evaluating the diagnostic performance of biomarkers, taking into account both sensitivity and specificity across a range of cut-off values. In the context of pyoderma in dogs, where superficial and deep forms can occur with overlapping clinical and laboratory features, ROC analysis may provide additional insight into the potential utility of serum IL-8 concentrations as a supportive diagnostic parameter. Based on these findings, a receiver operating characteristic (ROC) analysis was performed, yielding an area under the curve (AUC) of 0.74. An IL-8 serum concentration of 11.0 ng/mL provided the optimal cut-off value for differentiating superficial from deep pyoderma, with a sensitivity of 0.71 and a specificity of 0.79 (Figure 3).

### 3.6. Defb2—Serum Concentrations

**Figure 4:**
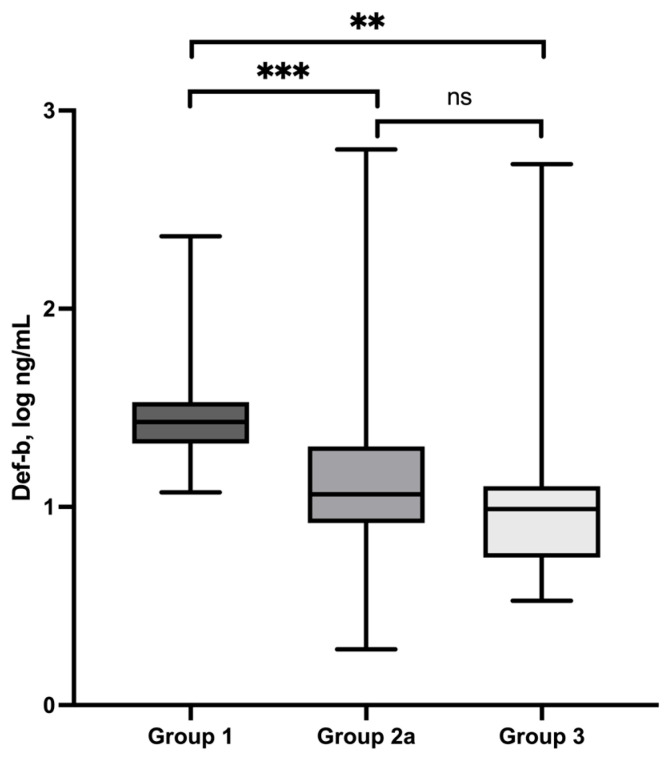
Defb2 serum concentrations (log) of control group (group 1), dogs with superficial pyoderma (group 2a) and deep pyoderma (group 3) and their statistical comparison. ns = not significant, ** 0.0025, *** 0.0003.

Defensin beta-2 (Defb2) serum concentrations were measured in 35 dogs of the control group (Group 1). The median concentration was 26.8 ng/mL (min/max: 11.9–231.9 ng/mL) (Table 1). Dogs with superficial pyoderma (Group 2a) showed lower Defb2 serum concentrations, with a median value of 11.6 ng/mL (min/max: 1.9–635.8 ng/mL). In dogs with deep pyoderma (Group 3), the median Defb2 serum concentration was 9.7 ng/mL (min/max: 3.4–536.9 ng/mL). Compared to healthy controls, Defb2 concentrations were significantly reduced in both pyoderma groups (superficial pyoderma: *p* = 0.0003; deep pyoderma: *p* = 0.0025). No statistically significant difference was observed between dogs with superficial and deep pyoderma (*p* > 0.53) (Figure 4). In follow-up measurements after clinical improvement (Group 2b; severity index: 14 → 2), Defb2 serum concentrations remained largely unchanged compared to baseline values, with a median concentration of 11.7 ng/mL (min/max: 6.4–97.7 ng/mL). No statistically significant difference between baseline and follow-up measurements was detected (*p* = 0.8).

### 3.7. Biomarker Ratios in Relation to Severity and Cytological Scores

The relationships between individual serum biomarkers, as well as calculated biomarker ratios, and the clinical severity index were investigated. No significant correlation was identified between any single biomarker and the severity of pyoderma as assessed by the severity index. In contrast, comparison with the cytological score revealed a relevant association with the biomarker ratio IL-8/procalcitonin. This ratio showed a moderate and statistically significant correlation with the cytological score (Spearman *p* = 0.037).

## 4. Discussion

Pyoderma is a common bacterial skin disease in dogs and is most frequently caused by staphylococcal species [16]. In the present study, *Staphylococcus pseudintermedius* was identified in all cases of superficial pyoderma, supporting its role as the primary pathogen in this condition. In contrast, dogs with deep pyoderma consistently showed a polymicrobial infection pattern, which is in accordance with previous reports [26]. Based on these considerations, it can be hypothesized that the depth of infection and bacterial complexity may influence the systemic immune response, with deep pyoderma potentially exerting a stronger systemic effect than superficial disease. Such associations have previously been described for classical inflammatory markers, including C-reactive protein and leukocyte counts [22,27]. Against this background, the present study investigated the impact of superficial and deep pyoderma on selected serum biomarkers of inflammation and innate immunity, aiming to determine whether these markers reflect disease presence, severity, and treatment response.

Procalcitonin (PCT) is widely recognized as a biomarker associated with systemic bacterial infections and sepsis in human medicine [28]. Although its diagnostic value is best established in humans, recent veterinary studies indicate that increased serum PCT concentrations may also be observed in dogs with inflammatory and infectious diseases [29]. In the present study, dogs with both superficial and deep pyoderma exhibited significantly higher serum PCT concentrations compared to healthy controls, confirming previous findings. A wide interindividual variation in PCT concentrations was observed within the diseased groups. While no statistically significant difference was detected between superficial and deep pyoderma, the numerically higher median PCT concentration in dogs with deep pyoderma may suggest a greater degree of systemic inflammatory involvement. Overall, these results support the concept that bacterial skin infections in dogs should not be regarded solely as localized disorders, but may, in selected cases, be associated with a measurable systemic inflammatory response.

Interleukin-8 (IL-8, also known as CXCL8) is a pro-inflammatory chemokine produced by macrophages, epithelial cells, and endothelial cells and plays a central role in neutrophil recruitment and activation during inflammatory responses. Previous studies in dogs with atopic dermatitis reported significantly increased plasma IL-8 concentrations compared to healthy controls, and IL-8 levels were shown to correlate with disease severity in otitis externa associated with atopic dermatitis [30,31]. Experimental studies further demonstrated that recombinant human IL-8 induces neutrophil migration in canine skin, underscoring its biological relevance in cutaneous inflammation [32]. Additional studies confirmed IL-8 expression in epithelial and fibroblastic cells in response to inflammatory, allergic, and bacterial stimuli [33].

In line with these reports, the present study demonstrated markedly elevated IL-8 serum concentrations in dogs with both superficial and deep pyoderma compared to healthy controls. Notably, IL-8 concentrations did not significantly decrease following clinical improvement, indicating persistent inflammatory activation despite apparent resolution of clinical signs. This finding suggests that IL-8 reflects ongoing neutrophil-mediated inflammatory processes that may outlast visible skin healing and are not adequately captured by clinical severity scoring alone.

Although dogs with superficial and deep pyoderma showed overlapping IL-8 serum concentrations, ROC analysis was performed to further explore the discriminatory potential of IL-8 beyond group-wise comparison of median values. As illustrated in Figure 2, IL-8 concentrations in both groups largely ranged between approximately 7 and 13 ng/mL, which makes visual discrimination based on absolute values challenging. This overlap explains why no statistically significant difference between the two groups was detected and underscores the limitations of relying solely on descriptive statistics.

ROC analysis allows evaluation of diagnostic performance across the full spectrum of observed values rather than at a single comparison point. The resulting AUC of 0.74 indicates a moderate ability of IL-8 serum concentrations to differentiate between superficial and deep pyoderma. The proposed cut-off value of 11.0 ng/mL was determined as the optimal balance between sensitivity (0.71) and specificity (0.79). Importantly, this cut-off should not be interpreted as a definitive threshold for diagnosis, but rather as a supportive parameter that may aid clinical decision-making when interpreted in conjunction with clinical findings and disease severity.

The substantial overlap in IL-8 concentrations between superficial and deep pyoderma reflects the shared inflammatory nature of both conditions and highlights that IL-8 alone is unlikely to serve as a standalone biomarker for depth differentiation. Nevertheless, the ROC-based approach demonstrates that IL-8 may still provide incremental diagnostic information at the individual level, which is not readily apparent from visual inspection of concentration ranges alone.

β-Defensins represent a key component of the first line of immunological defense in dogs with inflammatory skin diseases. Beyond their direct antimicrobial activity, β-defensins modulate immune responses by promoting the recruitment of immune cells such as neutrophils and macrophages [34]. To date, no studies have specifically investigated serum β-defensin concentrations in dogs with pyoderma. However, studies examining antimicrobial peptide expression in canine skin infections and atopic dermatitis have demonstrated that canine β-defensin 103 is detectable in both healthy and diseased skin without significant differences between infected and non-infected sites [35]. Increased expression and secretion of antimicrobial peptides have also been described in atopic dermatitis, particularly in response to superficial skin injury or infection [36].

In contrast to these tissue-level observations, the present study revealed significantly reduced serum concentrations of defensin beta-2 (Defb2) in dogs with superficial and deep pyoderma compared to healthy controls. This reduction was independent of disease depth and remained unchanged during follow-up examinations despite clear clinical improvement. Persistently decreased Defb2 serum concentrations may indicate increased consumption or sequestration of defensins at sites of inflammation. A disease-associated reduction in β-defensin-2 has also been described in other inflammatory conditions in dogs. In a study by Neumann et al. [29], dogs with acute diarrhea exhibited significantly lower β-defensin-2 concentrations compared to healthy controls, supporting the concept of altered defensin availability during active inflammatory disease. Although β-defensin-2 was assessed in fecal samples in that study, the findings are consistent with the hypothesis that defensins may be locally utilized or redistributed rather than systemically depleted through reduced production.

Similar to procalcitonin, Defb2 serum concentrations did not normalize following clinical remission. This lack of dynamic change suggests that systemically circulating inflammatory mediators may persist longer than clinical signs of disease, reflecting a delayed resolution of systemic immune activation relative to local skin healing. Collectively, these findings emphasize that inflammatory processes at the systemic level do not necessarily resolve in parallel with clinical improvement.

Although none of the individual serum biomarkers demonstrated a significant correlation with the clinical severity index, the evaluation of biomarker ratios provided additional insights into inflammatory activity. In particular, the ratio of IL-8 to procalcitonin (IL-8/PCT) showed a moderate but statistically significant correlation with the cytological score. This observation highlights an important distinction between clinical severity scores, which primarily reflect visible lesions, and cytological scores, which more directly represent cellular inflammatory activity and bacterial burden.

Interleukin-8 reflects local neutrophilic activation, whereas procalcitonin serves as an indicator of systemic bacterial inflammation. The IL-8/PCT ratio may therefore integrate local and systemic components of the inflammatory response, explaining its closer association with cytological findings than with clinical severity assessments. Receiver operating characteristic analysis supported the potential value of this combined approach, with an IL-8/PCT cut-off value of approximately 1.25 achieving a moderate discriminative performance for identifying dogs with high cytological scores (AUC = 0.73). While this accuracy does not support the use of the ratio as a standalone diagnostic tool, it suggests that biomarker ratios may serve as useful adjuncts in the evaluation of inflammatory activity in canine pyoderma.

Comparable observations have been reported in other inflammatory conditions in dogs. Neumann et al. demonstrated that combined biomarker approaches incorporating procalcitonin and β-defensin-2 improved disease characterization compared to individual markers alone [29]. Increased IL-8 expression has likewise been described in canine pyoderma and other inflammatory skin diseases [37]. Conversely, studies evaluating procalcitonin as a single biomarker in canine bacterial diseases have highlighted its limited diagnostic value when used in isolation [38]. Taken together, these findings underscore the complexity of inflammatory skin diseases and illustrate that no single systemic biomarker sufficiently reflects disease activity. Composite biomarker approaches, interpreted alongside cytological and clinical assessments, may therefore provide complementary and clinically relevant information.

## 5. Conclusions

In summary, the present study demonstrates that inflammatory skin diseases in dogs, particularly superficial and deep pyoderma, are associated with significant alterations in systemic inflammatory biomarkers, including procalcitonin, interleukin-8, and defensin beta-2. These findings support the concept that bacterial skin infections in dogs should not be regarded solely as localized conditions but may be accompanied by a measurable systemic inflammatory response.

While elevated concentrations of procalcitonin and interleukin-8 and reduced levels of defensin beta-2 were consistently observed in dogs with pyoderma compared to healthy controls, none of the individual biomarkers reliably reflected clinical disease severity or treatment response. This discrepancy between biomarker concentrations and clinical improvement indicates that the resolution of systemic inflammatory processes may lag behind visible clinical healing.

The analysis of biomarker ratios, particularly IL-8/procalcitonin, provided additional information and showed a moderate association with cytological inflammatory activity, suggesting that combined biomarker approaches may offer complementary value when interpreted alongside clinical and cytological findings. However, the results do not support the use of the investigated biomarkers as standalone diagnostic or prognostic tools.

From a clinical perspective, these findings highlight the importance of cautious interpretation of serum biomarkers and suggest that clinical improvement alone may not fully reflect the resolution of underlying inflammatory processes. This may have implications for therapeutic decision-making and treatment duration in individual cases.

The relatively small sample size, particularly in the deep pyoderma and follow-up groups, represents a limitation of the present study. Future investigations with larger patient cohorts and extended follow-up periods are warranted to further elucidate the diagnostic and clinical relevance of systemic inflammatory biomarkers in canine pyoderma. Another limitation was that biomarkers were measured only after clinical improvement. Further measurements should have been taken to determine how long serum levels of biomarkers remain elevated.

## Figures and Tables

**Table 1:** Comparison of serum concentrations (median, min-max) and statistical significance.

Group	PCT (ng/mL)	IL-8 (ng/mL)	Def (ng/mL)
Group 1	2.2 (0.5–9.1)	0.06 (0.02–0.19)	26.8 (11.9–231.9)
Group 2a	4.7 (3.0–7228.0)	10.0 (6.8–12.6)	11.6 (1.9–635.8)
Stat. comparison 1 vs. 2a	*p* < 0.0001	*p* < 0.0001	*p* = 0.0003
Group 2b	4.1 (3.1–7244.0)	10.3 (7.9–16.2)	11.7 (6.4–97.7)
Stat. comparison 2a vs. 2b	n.s. (*p* = 0.08)	n.s. (*p* = 0.48)	n.s. (*p* = 0.8)
Group 3	10.7 (3.6–1061.0)	11.9 (7.1–15.9)	9.7 (3.4–536.9)
Stat. comparison 1 vs. 3	*p* < 0.0001	*p* < 0.0001	*p* = 0.0025
Stat. comparison 2a vs. 3	n.s. (*p* > 0.4)	n.s. (*p* = 0.07)	n.s. (*p* > 0.53)

n.s. = not significant. All values = median (min–max).

## Data Availability

The original contributions presented in this study are included in the article. Further inquiries can be directed to the corresponding author.

## Abbreviations

AUC	Area Under the Curve
CRP	C-reactive protein
CXCL8	C-X-C motif chemokine ligand 8
CXCR1	C-X-C chemokine receptor 1
CXCR2	C-X-C chemokine receptor 2
CV	Coefficient of variation
Defb2	Defensin beta-2
EDTA	Ethylenediaminetetraacetic acid
ELISA	Enzyme-linked immunosorbent assay
IgG	Immunoglobulin G
IL-1	Interleukin-1
IL-6	Interleukin-6
IL-8	Interleukin-8
JAK3	Janus kinase 3
MALDI-TOF MS	Matrix-assisted laser desorption/ionization time-of-flight mass spectrometry
NSAIDs	Non-steroidal anti-inflammatory drugs
PCT	Procalcitonin
PBS	Phosphate-buffered saline
ROC	Receiver operating characteristic
SpsD	Staphylococcal protein D
SpsL	Staphylococcal protein L
TNF-α	Tumor necrosis factor alpha
